# Prognostic values of immune scores and immune microenvironment-related genes for hepatocellular carcinoma

**DOI:** 10.18632/aging.102971

**Published:** 2020-03-25

**Authors:** Peng-lei Ge, Shi-fang Li, Wei-wei Wang, Chun-bo Li, Yu-bin Fu, Zheng-kai Feng, Lin Li, Gong Zhang, Zhi-qiang Gao, Xiao-wei Dang, Yang Wu

**Affiliations:** 1 Department of Hepatobiliary and Pancreatic Surgery, The First Affiliated Hospital of Zhengzhou University, Zhengzhou, Henan Province, China; 2 Department of Pathology, The First Affiliated Hospital of Zhengzhou University, Zhengzhou, Henan Province, China

**Keywords:** immune score, tumour microenvironment, immune-related gene, hepatocellular carcinoma

## Abstract

It is crucial to grasp the characteristics of tumour immune microenvironment to improve effects of immunotherapy. In this study, the immune and stromal scores of 371 cases were calculated for quantitative analysis of immune and stromal cell infiltration in the tumour microenvironment of hepatocellular carcinoma (HCC). The weighted gene co-expression network analysis and protein–protein interaction network were analysed to identify immune microenvironment-related genes. The results showed that patients with high immune scores had a higher 4-year recurrence-free rate. TP53, CTNNB1, and AXIN1 mutations significantly varied with immune scores. In immune score-related modules analysis, Kyoto encyclopaedia of genes and genomes pathways and gene ontology terms were closely related to immune processes, tumorigenesis, and metastasis. Twelve new immune microenvironment-related genes were identified and had significantly positive correlations with seven immune checkpoint genes. In prognostic analysis, eleven immune microenvironment-related genes exhibited high expression, nine of which were validated in the GSE62232 dataset and were significantly associated with a good prognosis. Our findings suggest that calculating immune score and stromal score could help to determine tumour purity and immune cell infiltration in the tumour microenvironment. Nine immune microenvironment-related genes identified in this study had potential as prognostic markers for HCC.

## INTRODUCTION

Liver cancer is one of the most common malignancies and the second most frequent cause of cancer-related mortality worldwide [1]. Hepatocellular carcinoma (HCC), the predominant form of liver cancer, is highly malignant due to its insidious onset, rapid progression and metastasis. Due to the difficulty of early diagnosis, most patients with HCC have reached middle- or late-stage disease at diagnosis. Therefore, most have already missed the opportunity for surgery. Since radiotherapy and chemotherapy do not prolong overall survival (OS) in HCC [2], it is critical to explore other novel therapies, including targeted therapy and immunotherapy.

Immunotherapy is a very promising treatment for other cancer types such as melanoma and non-small cell lung cancer [3–4] and has the potential to change the landscape of therapy for malignancies in the future.

At present, immunotherapy-based immune checkpoint inhibitors are making big breakthroughs. Treatment of metastatic melanoma by monoclonal antibody destruction of immune checkpoints has become the new standard treatment and has replaced traditional chemotherapy. This may become the main therapy for other malignant tumours in the future [5]. However, due to the complexity of immunotherapy and tumour heterogeneity, this method is only effective for some patients. To match patients suffering from cancers to new promising treatments or clinical trials, genomic analysis of tumor samples could be performed [6]. Thus, it is important to determine the characteristics of the tumour immune microenvironment and to identify patients eligible for immunotherapy to improve the effects of immunotherapy [7].

The tumour microenvironment consists of immune cells, stromal cells, endothelial cells, inflammatory mediators, and extracellular matrix molecules, among which immune cells and stromal cells are the two main types of non-tumour components [8]. The purity of tumour tissue is an important feature of tumour heterogeneity. The prognostic evaluation is valuable, and these non-tumour cells dilute the purity of tumour cells, which have an important role in tumour growth. By analysing these non-tumour cells, especially immune cells and stromal cells in the tumour microenvironment, clinicians can more accurately understand the purity and immunological characteristics of tumours, and may implement new approaches to personalized medicine.

The immune and stromal scores calculated based on the ESTIMATE (Estimation of STromal and Immune cells in MAlignant Tumor tissues using Expression data) algorithm can promote the quantitative analysis of immune and matrix components in tumours [9]. This algorithm had been applied in breast cancer [10], colon cancer [11], and glioblastoma [12], in which immune and stromal scores were calculated by analysing specific gene expression profiles of immune and stromal cells to predict non-tumour cell infiltration.

In this study, we took advantage of data from The Cancer Genome Atlas (TCGA) to analyse: (1) the content of immune cells and stromal cells infiltrating the tumour microenvironment of HCC; (2) the relationship between immune scores and prognosis; (3) the relationship between immune scores and *TP53*, *CTNNB1*, and *AXIN1* mutations; and (4) immune microenvironment-related genes and their impact on prognosis.

## RESULTS

### Relationship of ESTIMATE scores with immune infiltration

**Figure 1:**
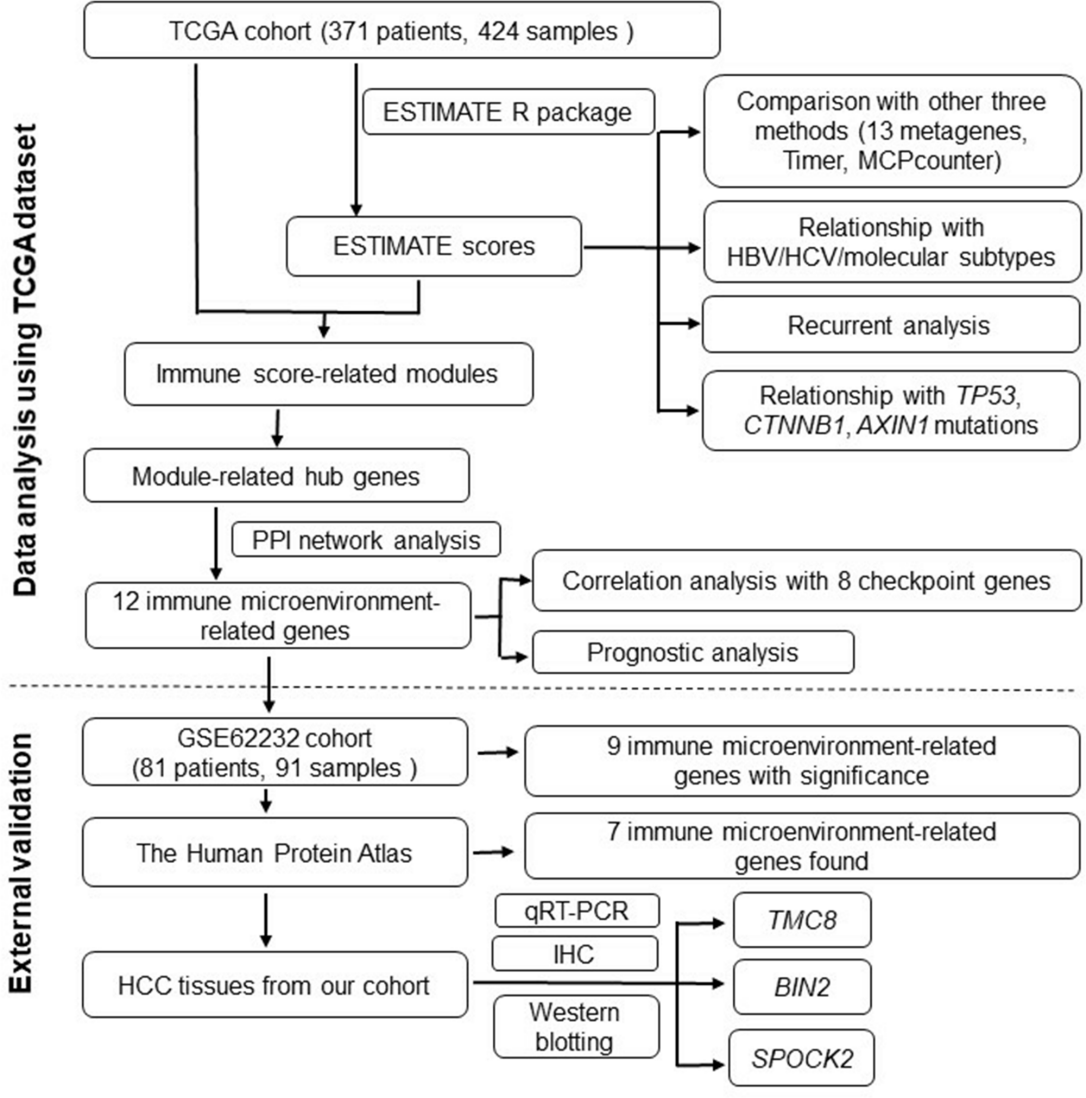
**Flowchart describing the procedure of analyzing and validating the prognostic values of immune scores and immune microenvironment-related genes.**

**Figure 2:**
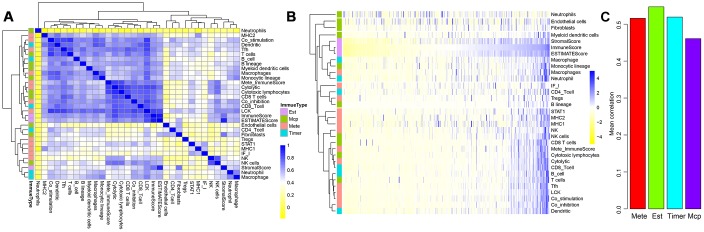
**Correlations between three ESTIMATE scores and other types of immune-related scores.** (**A**) Clustering heat map analyzed Spearman’s rank correlation coefficient. (**B**) Hierarchical clustering heat map using correlation to calculate distance. (**C**) Mean correlations of four methods using to calculating immune scores.

A flowchart of the analysis procedure for this study is shown in Figure 1. We used four different methods to analyse the correlation of scores of all immune-related cell types. As shown in Figure 2A, the mean correlation of different immune cells was larger than 0.5. The ten most correlated immune cell scores with other scores were LCK (R=0.69), Co_stimulation (R=0.62), dendritic (R=0.62), Tfh (R=0.61), Co_inhibition (R=0.61), cytolytic (R=0.6), CD8_Tcell (R=0.59), ImmuneScore (R=0.59), ESTIMATEScore (R=0.58), and cytotoxic lymphocytes (R=0.57). The concentrations of immune-related cells calculated with different methods had a certain consistency. In hierarchical clustering heat maps of various scores (Figure 2B), we found immune cell scores in each sample by different methods were also consistent.

We further tested the average correlations between the immune scores calculated by the four methods and other types of scores (Figure 2C). It could be seen that immune scores calculated by ESTIMATE that were larger than 0.54 had the highest average correlation than that using the other three methods. This indicated that ImmuneScore, StromalScore, and ESTIMATEScore calculated by the ESTIMATE method were closely related to components of immune cells in the tumour microenvironment.

### Relationship between ESTIMATE immune scores and HBV/HCV/molecular subtypes

**Figure 3:**
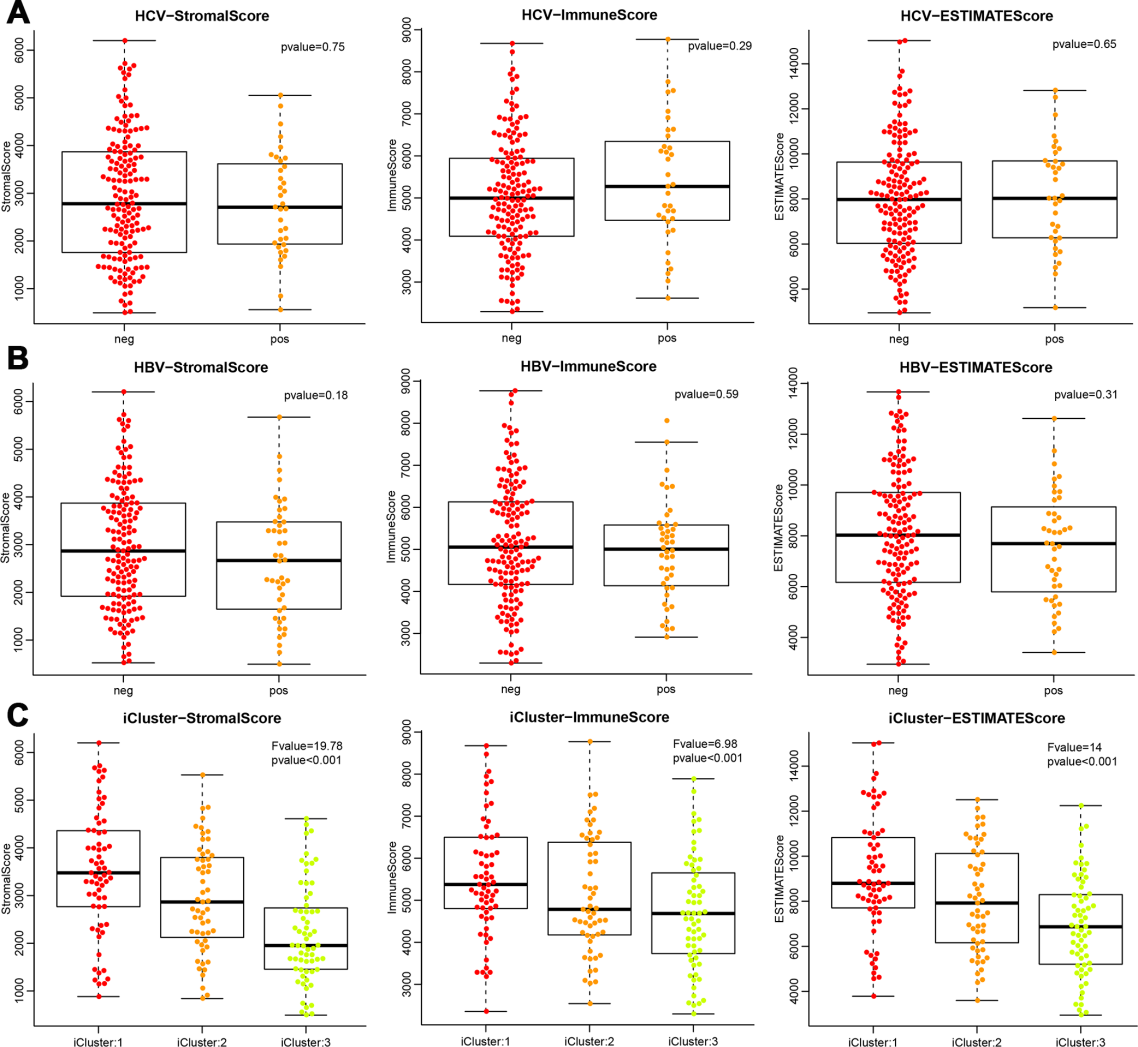
Differences of the three ESTIMATE immune scores respectively in (**A**) HCV-positive and -negative patients, (**B**) HBV-positive and -negative patients, (**C**) three molecular subtypes based on mRNA expression profiles.

Based on the three scores generated by the ESTIMATE algorithm, we analysed the relationship between immune scores and HBV/HCV/molecular subtypes that had been reported in previous comprehensive genomic analysis of liver cancer [13] (Supplementary file 1). As shown in Figure 3, we could see that HBV and HCV factors had no significant effect on ImmuneScore, StromalScore, and ESTIMATEScore (all *P*>0.05). However, the three scores in the three molecular types of expression profiles were significantly different, and iCluster3 had the lowest score (all *P*<0.001), suggesting a difference in the immune scores among the molecular subtypes in liver cancer.

### Recurrent analysis of immune scores

**Figure 4:**
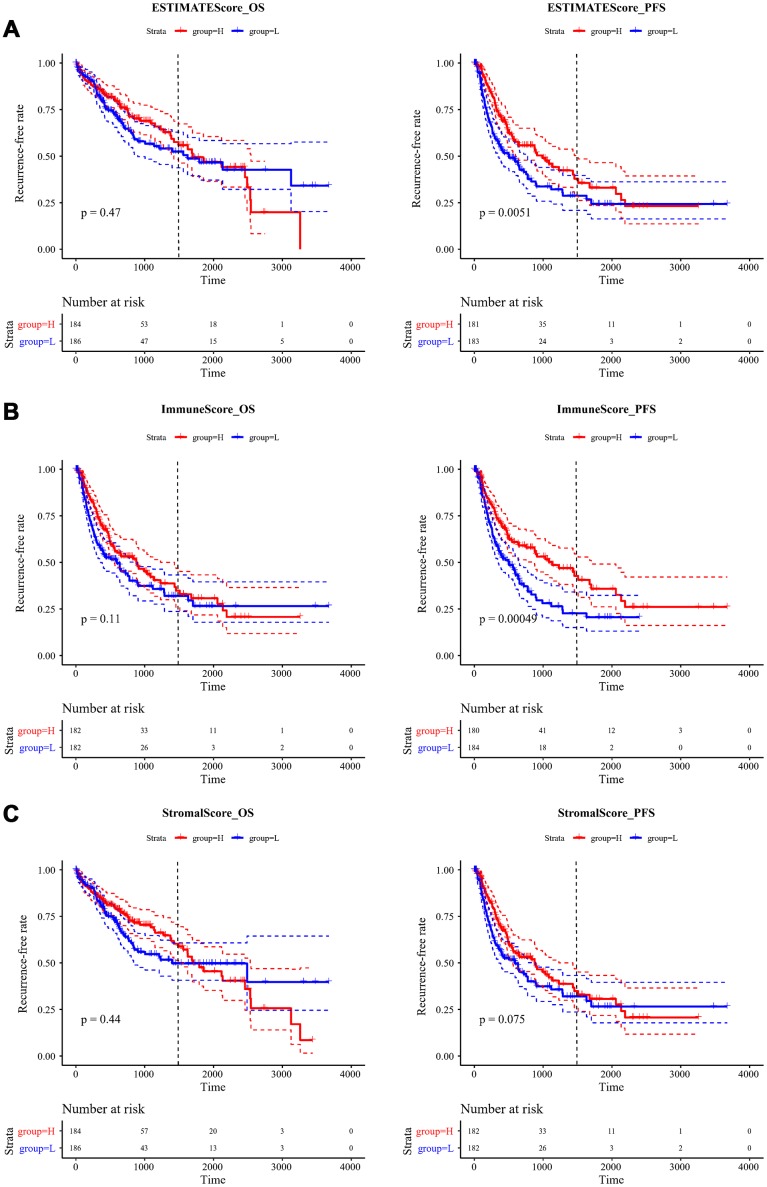
**Kaplan-Meier curve for recurrent analysis of overall survival time and progression-free survival time by immune scores.** (**A**) ESTIMATEScore, (**B**) ImmuneScore, (**C**) StromalScore. H (red solid line) and L (blue solid line) respectively represented high-score group and low-score group. The red dotted line and blue dotted line respectively represented upper and lower limit of 95% confidence intervals. The vertical dotted line was at 4 years. OS, overall survival time; PFS, progression-free survival time.

To observe the relationship between ESTIMATE scores and recurrence, we classified all the samples into high- and low-score subgroups according to the median ESTIMATE immune score, then the Kaplan-Meier method was used for recurrent difference analysis. As shown in Figure 4A–4C, patients in the high-score group had a higher 4-year recurrence-free rate than the low-score group, especially for progression-free 4-year survival, indicating that the immune scores were potential predictive recurrent markers.

### Relationship between ESTIMATE immune scores and *TP53*, *CTNNB1*, and *AXIN1* mutations

**Figure 5:**
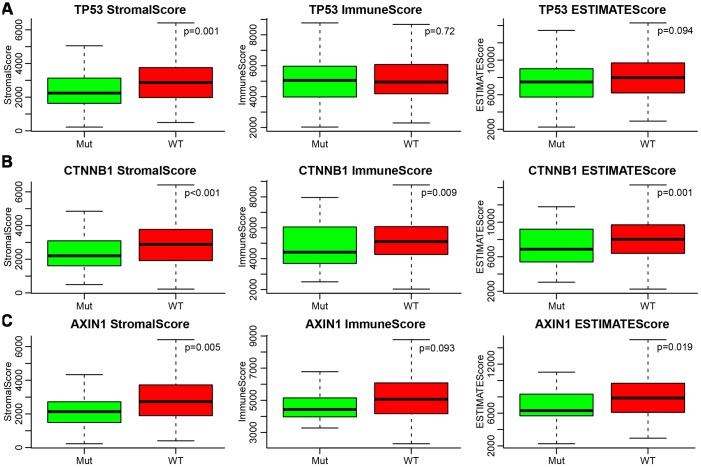
(**A**–**C**) Relationship between mutations of TP53, CTNNB1, AXIN1 and ESTIMATE’s immune scores. Mut, mutation group. WT, wild type group.

Many previous reports indicated that *TP53*, *CTNNB1* (β-catenin), and *AXIN1* mutations are closely related to liver cancer development. Therefore, we analysed the relationship between mutations of these three genes and ESTIMATE’s immune scores. Firstly, the mutation data of *TP53*, *CTNNB1*, and *AXIN1* were extracted from SNP data treated by Mutect in TCGA. Then, the relationship between the immune scores of the mutant and non-mutant group divided by the three genes was analysed separately (Figure 5A–5C). It could be seen that StromalScore had significant differences among all three of the mutated genes, with the mutation group being smaller than wild-type group (*p*=0.001 for *TP53*, *p*<0.001 for *CTNNB1*, *p*=0.005 for *AXIN1*). ImmuneScore was significantly lower in the *CTNNB1* mutant group than in the wild-type group, while ESTIMATEScores were significantly lower in the *CTNNB1* and *AXIN1* mutant groups than in the wild-type group.

### Immune score-related module analysis

As shown in Supplementary Figure A, clustering analysis of samples was performed. Three hundred and ninety-six samples were finally obtained after excluding the outliers. To ensure it was a scale-free network, we chose β=12 (Supplementary Figure B, C). Finally, a total of 15 modules was obtained (Supplementary Figure D). The grey module was a gene collection that could not be aggregated into other modules. The transcripts statistics of each module are shown in Table 1. It could be seen that 6,226 transcripts were assigned to 14 co-expression modules. The correlations between 15 module eigenvectors and the three immune scores were calculated (Supplementary Figure E). The blue and yellow modules had the highest correlation with the three immune scores, and the average correlation coefficients were 0.59 and 0.48, respectively. There were 319 and 142 genes in these two modules, totalling 461 genes.

**Table 1:** Numbers of genes included in 15 modules.

**Modules**	**Genes**
black	97
blue	319
brown	236
cyan	32
green	124
greenyellow	44
grey	5577
magenta	57
pink	90
purple	44
red	104
salmon	34
tan	44
turquoise	4859
yellow	142

**Figure 6:**
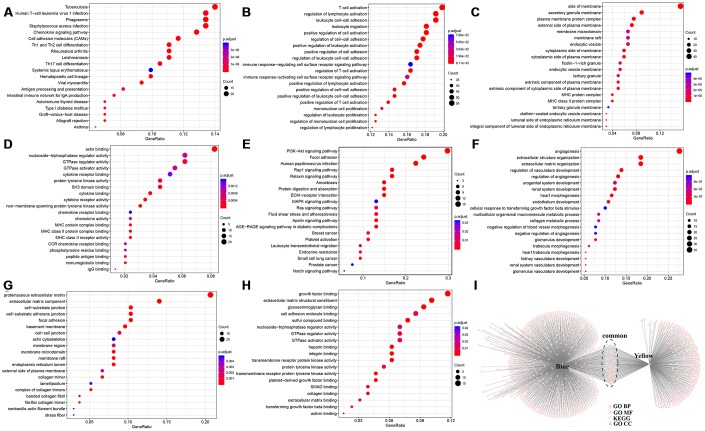
**Enrichment analysis of genes in modules most relevant to immune scores.** Top 20 (**A**) KEGG pathway, (**B**) GO BP, (**C**) GO CC, and (**D**) GO MF enriched by Blue module. Top 20 (**E**) KEGG pathway, (**F**) GO BP, (**G**) GO CC, and (**H**) GO MF enriched by Yellow module. (**I**) Intersections of KEGG pathway and GO Term enriched by Blue and Yellow modules. BP, biological processe. CC, cell component. MF, molecular function.

The functions of genes in the modules most relevant to the immune score were enrichment analysed (Supplementary file 2). The blue module was enriched in 85 KEGG pathways, 712 biological processes, 75 cell components, and 53 molecular functions. Meanwhile, the yellow module was enriched in 61 KEGG pathways, 280 biological processes, 35 cellular components, and 29 molecular functions. The most significant of the top 20 KEGG pathways and GO terms of the blue module are shown in Figure 6A–6D. We determined that: the KEGG pathways were mainly related to inflammation, biological processes were mainly related to T cell activation, cell compositions were mainly enriched in MHC class II protein complex, MHC protein complex, and other components, and molecular functions were mainly enriched in cytokine receptor activity, MHC class II receptor activity, and other functions. These pathways and GO terms were closely related to the immune process.

The top 20 KEGG pathways and GO terms of the yellow module are shown in Figure 6E–6H. It could be seen that: the module-enriched KEGG pathways were mainly related to focal adhesion and the PI3K–AKT signalling pathway, biological processes were mainly related to angiogenesis process, cell compositions were enriched in proteinaceous extracellular matrix, cell–substrate junctions, and other components, and molecular functions were mainly enriched in functions such as growth factor binding and transmembrane receptor protein kinase activity. These pathways and GO terms were closely related to tumorigenesis and metastasis.

Further analysis of the relationship between the two modules enriched in KEGG pathways and GO terms is shown in Figure 6I. The blue and yellow modules had only a small number of common intersections, which suggested that genes in the two modules performed different functions.

### Module-related hub gene screening

**Figure 7:**
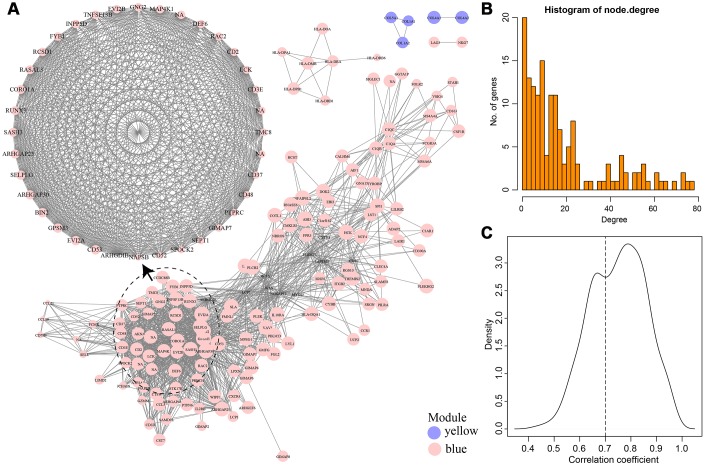
(**A**) Weight co-expression network of genes in Blue and Yellow modules. (**B**) The degree distribution of the network. (**C**) The correlation coefficient between genes and modules.

To select genes related to immune score, we calculated the weight of the relationship between genes in the two modules. We selected an inter-gene weight threshold greater than 0.2, and finally obtained a weight co-expression network of the genes in the two modules. As shown in Figure 7A, the network consisted of 155 nodes and 1,475 edges, including 150 blue module genes and five yellow module genes. It could be seen that more relevant the module was, the closer the genes were to other genes in the network.

The largest network module had 36 genes, and the degree distribution of the network was further analysed. As shown in Figure 7B, the degree of most nodes was small, and a small number of nodes were large, which was consistent with the characteristics of biological networks. We calculated the correlation coefficient between genes and modules in the two co-expression modules (Figure 7C). It was more than 0.7 for most genes, indicating that genes in the module had high expression similarity. We selected 36 genes as immune microenvironment-related genes, which belonged to the largest network module, and a correlation coefficient greater than 0.7 (Supplementary file 3). Ten LCK metagenes and 26 blue module genes were included.

### Network interaction analysis of immune microenvironment genes

**Figure 8:**
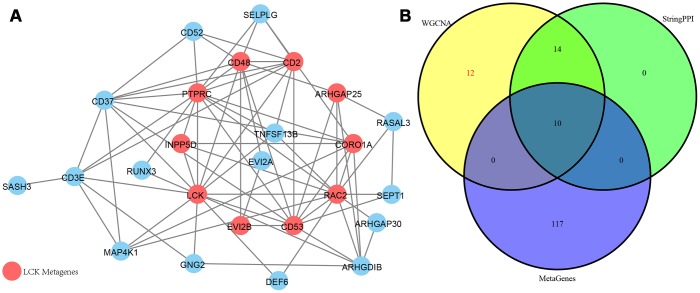
(**A**) PPI network of the immune microenvironment genes. Red circle was LCK Metagenes. Light blue circle represented genes belonged to Blue module. (**B**) Venn diagram showing the number of genes in WGCNA, StringPPI and LCK Metagenes. WGCNA was from co-expression network. StringPPI was from direct interaction with LCK Metagenes in protein interaction network. MetaGenes was a merger of 13 set of MetaGenes.

As shown in Figure 8A, a total of 74 edges and 25 nodes were obtained. Fourteen (93.3%) of the genes interacted directly with LCK metagenes in the PPI network, except *SASH3*. We further compared genes that interacted directly with LCK metagenes in the PPI network with co-expressed genes (Figure 8B). Finally, we screened 12 genes that were co-expressed with immune scores and did not interact directly with LCK metagenes, which might be new genes associated with the immune microenvironment (Table 2).

**Table 2:** 12 potential immune microenvironment genes.

**ENSG**	**Symbol**	**corr.R**	**Module**
ENSG00000131401	NAPSB	0.706393	blue
ENSG00000082074	FYB1	0.869508	blue
ENSG00000110934	BIN2	0.937472	blue
ENSG00000179144	GIMAP7	0.775353	blue
ENSG00000167895	TMC8	0.801026	blue
ENSG00000277734	TRAC	0.855262	blue
ENSG00000211772	TRBC2	0.813236	blue
ENSG00000107742	SPOCK2	0.759702	blue
ENSG00000213654	GPSM3	0.939374	blue
ENSG00000211753	TRBV28	0.796667	blue
ENSG00000198771	RCSD1	0.870759	blue
ENSG00000122122	SASH3	0.896006	blue

### Relationship between 12 new immune microenvironment genes and immune checkpoint genes

We further analysed the correlation between the 12 new immune microenvironment genes and eight immune checkpoint genes, including

**Figure 9:**
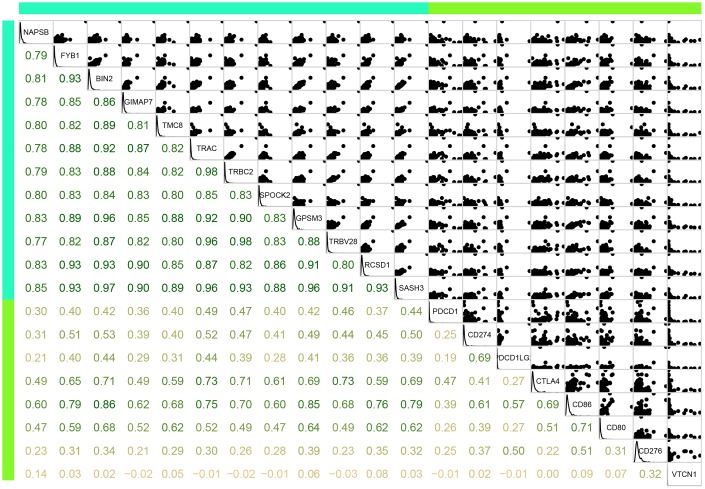
**Correlation between the 12 immune microenvironment genes (blue green bar) and 8 immune checkpoint genes (light green bar).** Lower panel was Pearson correlation coefficient between each gene. Upper panel was scatter plot of expressions between each gene. Diag panel was expression of each gene.

*PDCD1*, *CD274*, *PDCD1LG2*, *CTLA4*, *CD86*, *CD80*, *CD276*, and *VTCN1*. As shown in Figure 9, there were significantly positive correlations between the new immune microenvironment genes and seven immune checkpoint genes, except *VTCN1*. The average correlation coefficient was greater than 0.52, suggesting that these immune microenvironment genes might be potential targets for immunotherapy.

### Prognostic value of 12 immune microenvironment genes

**Figure 10:**
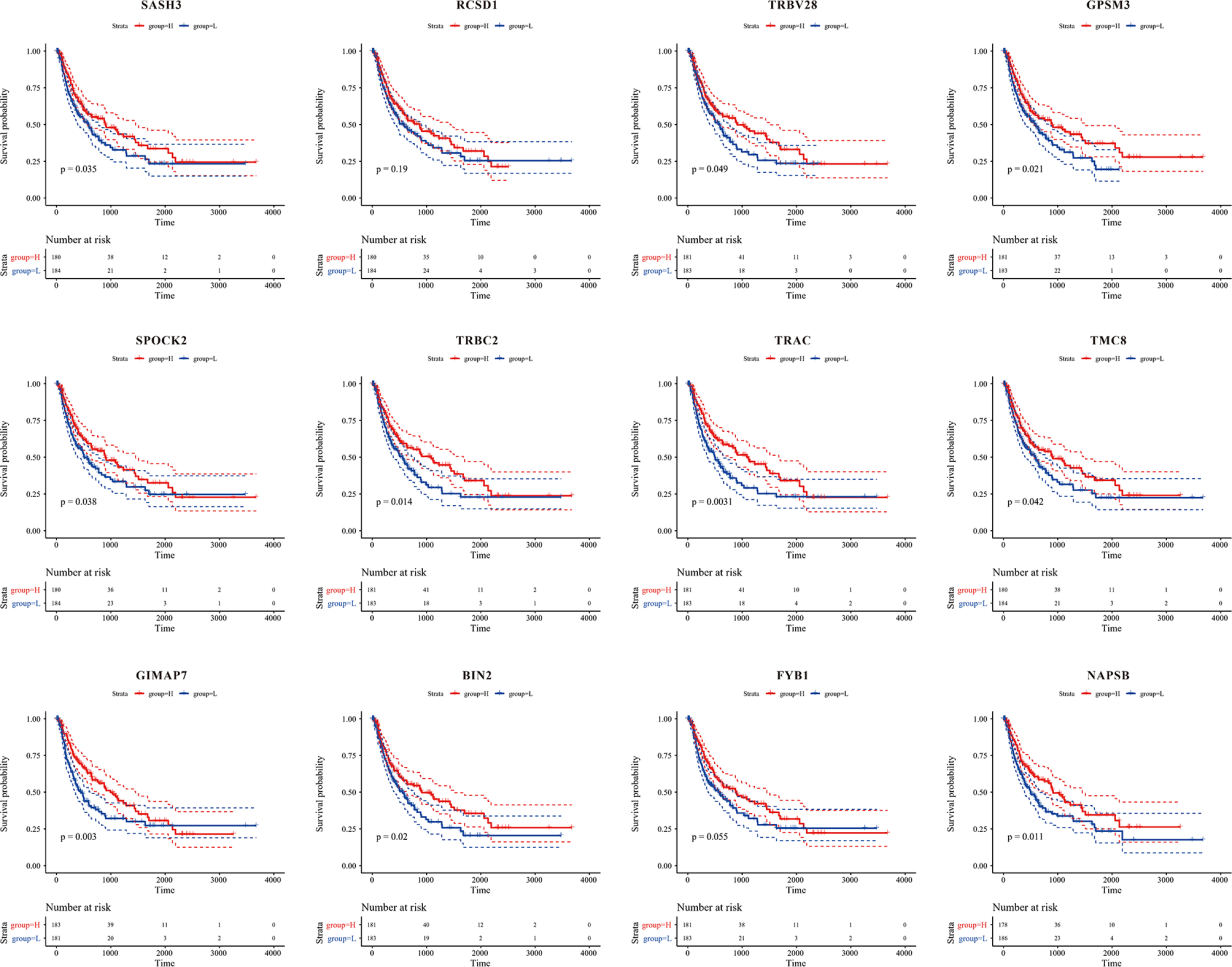
**Kaplan-Meier curve for prognostic analysis of the 12 immune microenvironment genes.** The red dotted line and blue dotted line respectively represented upper and lower limit of 95% confidence intervals of gene expression.

From Figure 10, we could see that, except for *RCSD1*, high expression of the other 11 genes was significantly associated with a good prognosis (*P*<0.05).

### External validation of the expression of the 12 immune-related genes and their relationship with immune scores

**Figure 11:**
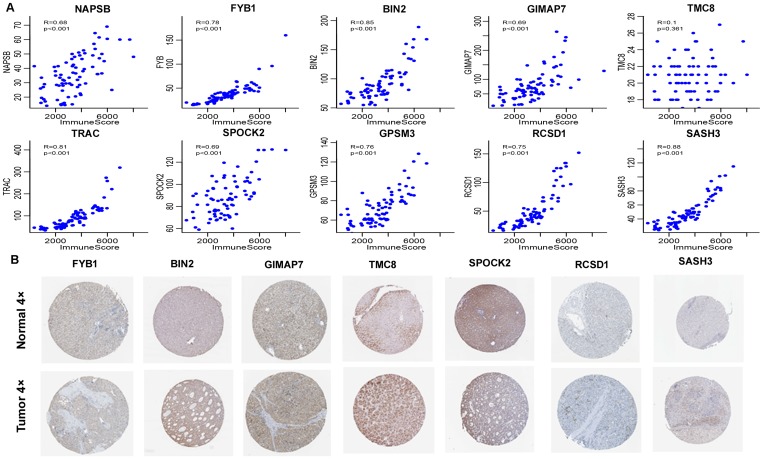
**External validation of 12 immune-related genes.** (**A**) Pearson correlation of expression and ImmuneScore using GSE62232 dataset. TRBC2 and TRBV28 were not detected. 9 genes, except TMC8, had significant correlation. Y-axis represented expression level of the genes in each sample. (**B**) Representative immunohistochemical staining images of 7 genes in normal liver tissue and HCC specimen. Images were taken from the Human Protein Atlas (https://www.proteinatlas.org). *TMC8* was strongly positive, *BIN2*, *GIMAP7*, *SPOCK2* were moderately positive, and *FYB1*, *RCSD1*, *SASH3* were weakly positive in HCC tissues relative to their expression levels in normal liver tissues.

In the GSE62232 dataset, two immune microenvironment gene expression profiles were not detected, so the other 10 genes were used to calculate their correlation with immune scores. As shown in Figure 11A, nine genes, except *TMC8*, had significant correlations with the immune score (*P*<0.001), which was consistent with the analysis in TCGA cohort.

In the validation of protein expression encoded by the genes using clinical specimens from Human Protein Profiles, we found that *TMC8* was strongly positive, *BIN2*, *GIMAP7*, and *SPOCK2* were moderately positive, and *FYB1*, *RCSD1*, and *SASH3* were weakly positive in HCC tissues relative to their expression levels in normal liver tissues (Figure 11B). However, *NAPSB*, *TRAC*, *TRBC2*, *GPSM3*, and *TRBV28* were not found on the website.

### Validation of expression of 3 immune-related genes in HCC tissues

**Figure 12:**
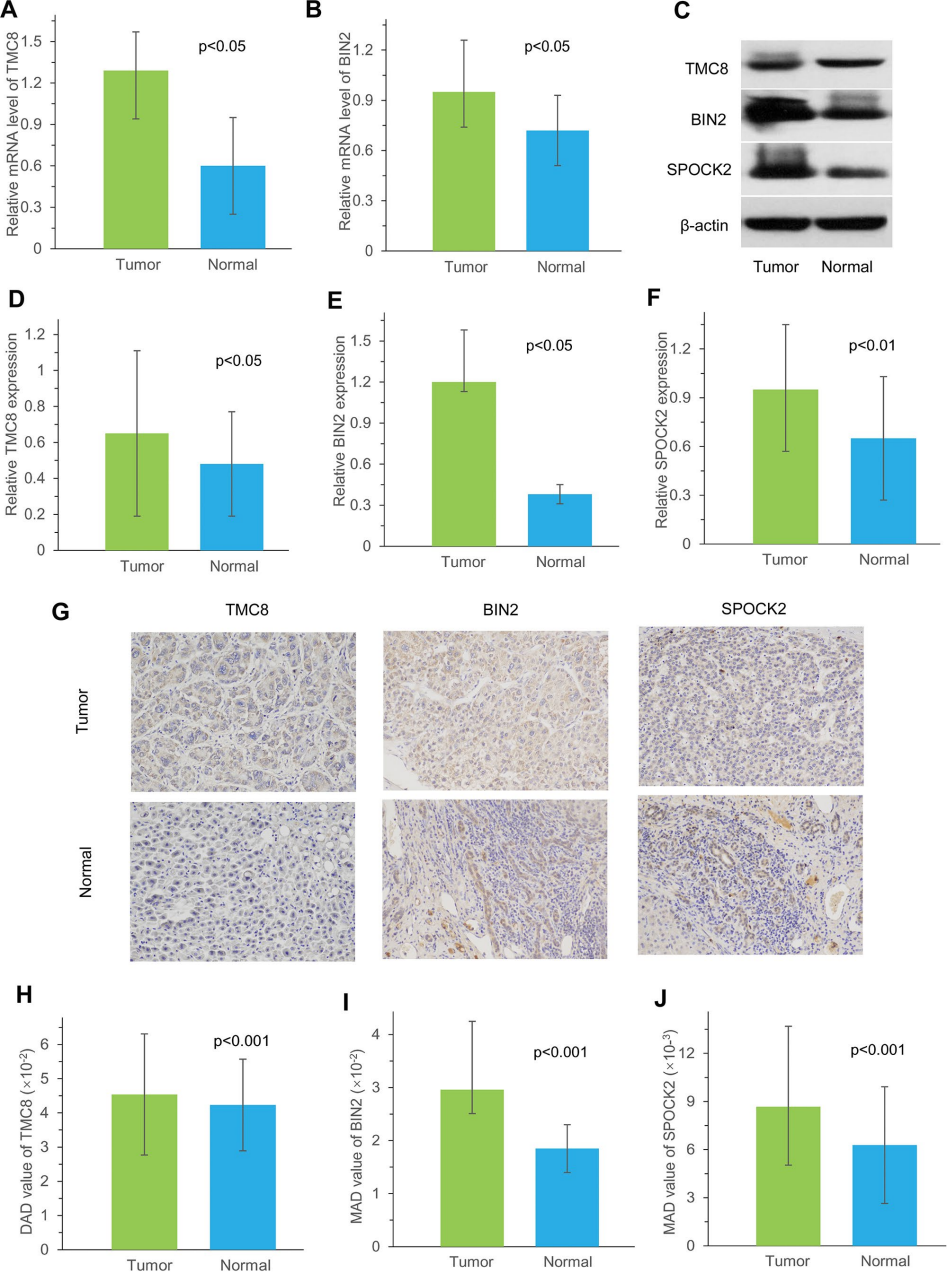
**Measurement of *TMC8*, *BIN2* and *SPOCK2* at mRNA and protein level in our cohort.** (**A**, **B**) Relative mRNA levels of *TMC8* and *BIN2* in 10 HCC samples were both overexpressed compared with matched normal samples by qRT-PCR. (**C**) Representative western blotting images showed protein expression of *TMC8*, *BIN2* and *SPOCK2* were overexpressed in HCC tissue than those in normal liver tissue. (**D**–**F**) Western blotting analysis demonstrated that mean greyscale of *TMC8*, *BIN2* and *SPOCK2* were all higher in 10 fresh-frozen HCC tissues compared with those in matched adjacent normal liver tissues. (**G**) Representative immunohistochemical staining images of *TMC8*, *BIN2* and *SPOCK2*, which were all mainly expressed in the cytoplasm. Original magnification: x200. (**H**–**J**) Mean protein expression of *TMC8*, *BIN2* and *SPOCK2* in 35 HCC were all significantly higher compared with those in adjacent non-tumour tissue by immunohistochemistry. MAD: mean areal density.

To further validate the results, qRT-PCR was applied to analyse the relative mRNA expression of *TMC8* and *BIN2* in fresh HCC and adjacent non-tumour tissues. The results showed that elevated expression of *TMC8* and *BIN2* relative mRNA in tumor tissues compared to adjacent normal tissues (Figure 12A–12B). Our western blotting analysis demonstrated that HCC tissues exhibited relative higher levels of *TMC8,* *BIN2* and *SPOCK2* protein expression than those in normal liver tissues (Figure 12C–12F). In addition, IHC analysis was also conducted to determine *TMC8,* *BIN2* and *SPOCK2* protein expression level in HCC. From the immunostaining, we could observe the positive immunoreactivity were primarily localized in the cytoplasm (Figure 12G). The three proteins were all up-regulated in tumor tissues compared with the adjacent normal liver tissues (Figure 12H–12J). These results all indicated that *TMC8,* *BIN2* and *SPOCK2* were overexpressed in HCC tissues.

## DISCUSSION

The tumour microenvironment is crucial for the occurrence, development and prognosis of tumours and is one of the main causes of tumour heterogeneity [14]. In cancer treatment, different strategies should be adopted for different tumour subtypes. Due to the heterogeneity of tumours, even the same treatment modalities often lead to quite different results for patients with the same pathological tumour subtypes. Immunotherapy has gained more attention as a very promising treatment and is being studied more deeply. As the two major non-tumour components in the tumour microenvironment, the composition of immune cells and stromal cells is of great value for the prognosis of tumours. Therefore, identifying the correct tumour immune subtype has a very important role in guiding the clinical treatment for tumours and monitoring prognosis.

The ESTIMATE algorithm uses immune genes and stromal genes to calculate tumour immune scores and stromal scores, respectively, which have been used to assess the immunological characteristics of gliomas [12]. It also reflects the purity of tumour cells. In this study, we applied various algorithms to test the correlation between the scores of immune-related cell types from HCC patients in TCGA database. In comparison, the ESTIMATE algorithm more accurately represented the tumour microenvironment than the other three methods. Understanding immune cell infiltration in the HCC microenvironment laid the foundation for the next analysis.

Chronic HBV and HCV infection are two major viral risk factors for HCC. A total of 22.4% patients displayed evidence of HBV infection in TCGA dataset, especially those of African ethnicity, younger age, and male sex. Approximately 17.9% patients presented with HCV infection, mainly Caucasian and black individuals [13]. Our analysis found no significant effect of the virus on the patients’ immune scores. However, when dividing HCC patients into three iClusters according to demographic, pathologic, and molecular features [13], we found that the three types of immune scores were significantly different (*P*<0.001). This suggested that patients’ immune characteristics were variable under different ethnic backgrounds, pathological types, and molecular characteristics.

In the predictive recurrent analysis, the three types of immune scores were divided into high and low scores. The 4-year recurrence-free rate was higher in the high immune score group than that in the low immune score group, especially for ImmuneScore and ESTIMATEScore. This indicated that the proportion of immune cells and tumour purity in the tumour microenvironment had an important effect on the prognosis of HCC. Patients with abundant immune cell infiltration in the microenvironment had better response to treatment and could predict the recurrent rate of patients for 4 years. Patients with preoperative low immune scores should receive a comprehensive therapeutic regimen that includes chemotherapy, targeted therapy, and immunotherapy after surgery to improve prognosis. For patients with high immune scores, we should mainly focus on administering different immunotherapies combined with conventional treatments to improve prognosis.

In previous studies, it was found that *TP53*, *AXIN1*, and *CTNNB1* were the three most frequently mutated genes that were closely related to the tumorigenesis and development of HCC, suggesting the wild-type genes have tumour suppressive roles [15–18]. *TP53* is a tumour suppressor gene, and its mutation is a recognized carcinogenic factor. Studies have found that *TP53* mutations are associated with the occurrence of liver cancer. *TP53* induces growth arrest or apoptosis of tumours depending on the physiological settings and cell type [19, 20]. Furthermore, *TP53* mutations in HCC patients are linked with worse clinical stage and prognosis [21]. Meanwhile, approximately 5%–19% of patients with HCC have *AXIN1* mutations [17]. AXIN1 can control the level of β-catenin and serve as a negative regulator of Wnt/β-catenin signalling. Overexpression of wild-type *AXIN1* could suppress the proliferation of HCC cells and accelerate their programmed cell death, which implies that AXIN1 is a therapeutic target in HCC [22]. Approximately 11%–41% of HCC harbours *CTNNB1*-activating mutations [17, 21]. β-catenin is an important component of the canonical Wnt signalling pathway. It anchors the actin cytoskeleton and might be responsible for communicating the contact inhibition signal that causes cells to halt division [19, 23]. We analysed the relationship between liver cancer immune scores and *TP53*, *AXIN1*, and *TP53* gene mutations, and found that StromalScore was significantly smaller in the *TP53* mutant group than in the wild group (*P*<0.05). The three immune scores were significantly smaller in the *CTNNB1* mutant group than that in the wild-type group, while in the *AXIN1* mutation group, StromalScore and ESTIMATEScore were smaller than in the wild-type group. This meant that the three mutated genes might affect the immune status and immune response of the liver cancer, and the immune score was related to the mutation of the related gene [24].

The assessment of molecular profiling combined with clinical data had identified personalized therapies and clinical trials for a large proportion of patients with cancer [6]. As a new promising treatment, studies on genetic aspects related to immunotherapy were urgently required. Through the calculation of immune scores, we could relatively accurately determine the tumour purity and immune cell infiltration in the tumour microenvironment. Finding new genes related to the immune microenvironment could help us analyse the immune status of patients more deeply and in a more targeted way.

We performed co-expression analysis based on expression profile data. The results revealed that the KEGG pathways enriched by the blue model were mainly related to inflammation. The biological process and cell component enrichment analysis revealed that it was mainly about immune processes. The results of enrichment analysis of the yellow module suggested that it was closely related to the occurrence and development of tumours. Subsequently, we found 12 genes associated with the HCC immune microenvironment.

To verify the accuracy of these genes, we analysed two external datasets: the GEO dataset and clinical immunohistochemistry specimens from Human Protein Profiles. By analysing the relationships between the 10 immune-related genes in the GEO dataset and immune scores, we found that nine of these genes, including *NAPSB*, *FYB1*, *BIN2*, *GIMAP7*, *TRAC*, *SPOCK2*, *GPSM3*, *RCSD1*, and *SASH3* had a high correlation with the immune score, which was consistent with our analysis in TCGA dataset. We also used data from Human Protein Profiles to demonstrate that the proteins encoded by seven immune-related genes were expressed at different degrees in HCC tissues, among which *TMC8*, *BIN2*, *GIMAP7*, and *SPOCK2* were strongly to moderately positive in tumours. The two external datasets verified the accuracy of the results, and these genes might serve as potential markers for the immune status of HCC.

As the main current tumour immunotherapy, immune checkpoint inhibitors could regulate the energy metabolism of tumour cells, the microenvironment, and tumour-specific immune responses [25]. The immune checkpoint is an immune molecule that regulates T cell immune response and is mainly distributed on the surface of antigen-presenting cells, T cells, and tumour cells [26]. Therefore, we verified the correlations between the tumour immune microenvironment and eight representative immune checkpoint genes, including *PDCD1*, *CD274*, *PDCD1LG2*, *CTLA4*, *CD86*, *CD80*, *CD276*, and *VTCN1*. The results showed that there was a significant positive correlation between immune microenvironment genes and all the immune checkpoint genes other than *VTCN1*. This suggested that these immune microenvironment genes might be potential immunotherapeutic targets.

To better understand the effects of immune and stromal cell-related genes on prognosis, we also analysed the tumour microenvironment-related genes with poor prognosis to explore the underlying regulatory mechanisms. We identified 12 immune microenvironment-related genes and revealed that patients with high expression of immune microenvironment genes except for *RCSD1* (*P*>0.05) would have a better prognosis (*P*<0.05). This also reflected to some extent that the immune microenvironment is an important component of immunotherapy. Detection of the immune microenvironment could help us better grasp the immune status of patients and might provide clinicians with more accurate treatment strategies.

To further validate the accuracy, we selected three immune-related genes, *TMC8*, *BIN2* and *SPOCK2*, to be evaluated using three different methods in our cohort recruited from the First Affiliated Hospital of Zhengzhou University. The results revealed the overexpression of *TMC8* and *BIN2* mRNA in HCC tissues. Furthermore, *TMC8*, *BIN2* and *SPOCK2* protein levels were also consistently higher expressed in tumor tissues than those in normal liver tissues.

In summary, we used the ESTIMATE algorithm to analyse the immune cell and stromal cell scores of the HCC microenvironment according to relative genes in TCGA database. Patients with high immune scores had a higher 4-year survival rate. Moreover, we revealed that mutations of *TP53*, *CTNNB1*, and *AXIN1*, having close relationships with the development of liver cancer, varied with various immune scores. We also applied the WGCNA to the analysis and identified 12 genes related to the immune microenvironment of HCC. Through the validation of an external dataset, GSE62232, we found that the results of nine genes were consistent with that in TCGA dataset, and had potential as prognostic markers for HCC. However, the results should be further validated using large-scale clinical data.

## MATERIALS AND METHODS

### Data from TCGA cohort

The transcriptome expression profiles and corresponding clinical information of HCC were downloaded from the Genomic Data Commons Application Programming Interface of TCGA (https://cancergenome.nih.gov/). Surgical resection samples were collected from patients who were diagnosed with HCC and did not receive prior adjuvant treatment for their disease. FPKM (fragments per kilobase million) data of RNA-Seq was first downloaded and then transferred to TPM (transcripts per million) expression data. The expression data also included single nucleotide polymorphism (SNP) expression data, containing 371 cases and 424 files as of February 2019.

### Data analysis

Four different algorithms were used to calculated the scores of related immune cells. The expression levels of 13 immune metagenes were listed, including ImmuneScore, LCK (lymphocyte-specific protein tyrosine kinase), TFH, Tregs, cytolytic, MHC1, MHC2, NK, macrophages, STAT1, IF_I, Co_stimulation, and Co_inhibition, which corresponded to various immune cell types and reflected various immune functions [10] (Supplementary file 2). Based on the gene expression levels of these metagenes, their scores were calculated using the median expression level of each gene in all the samples (Supplementary file 3).

There were large numbers of immune cell-specific genes highly expressed in the tumor microenvironment. The scores of six immune cell types of liver cancer, including B cells, CD4 T cells, CD8 T cells, neutrophils, macrophages, and dendritic cells, were downloaded from the Timer (https://cistrome.shinyapps.io/timer/) database, which used constrained least squares fitting on the informative immune signature genes to detect the immune cell infiltration in tumour tissue [27] (Supplementary file 4).

ESTIMATE is an algorithmic tool for predicting tumour purity, which uses the gene expression profiles of 141 immune genes and 141 stromal genes to generate ESTIMATE scores [9]. The presence of infiltrated immune cells and stromal cells in tumour tissues were calculated using related gene expression matrix data, represented by ImmuneScore and StromalScore, respectively (Supplementary file 5).

The abundance of eight immune cell types, including T cells, CD8 T cells, cytotoxic lymphocytes, NK cells, B lineage, monocytic lineage, myeloid dendritic cells, neutrophils, and other two types of cells, including endothelial cells and fibroblasts, in each sample were estimated with MCPcounter software (Supplementary file 6).

To explore the relationship of ESTIMATE scores with other immune scores, we analysed the scores of several immune-related cell types using the four different algorithms.

Relationship between ESTIMATE’s immune score and *TP53*, *CTNNB1*, and *AXIN1* mutations was also analysed.

### Mining immune score-related modules

To further explore prognostic markers related to the immune microenvironment of liver cancer, we obtained 423 expression profiles of all samples. More than 75% transcripts, totalling 11,803 TPM >1 and median absolute deviation greater than the median were selected in these samples. Firstly, hierarchical clustering analysis of the samples was undertaken, and outliers with a height over 100,000 were excluded. Distances between each transcript were calculated with Pearson’s correlation coefficient. WGCNA was constructed using the R package WGCNA.

The co-expression network conformed to a scale-free network, which was to say log(k) of the node with connection degree k was negatively correlated with log(P(k)) of probability of the node, and the correlation coefficient was greater than 0.85. To ensure it was a scale-free network, we chose β=12 (Supplementary Figure). Then, the expression matrix was converted into an adjacency matrix, which was converted into a topological matrix. Hierarchical clustering analysis of genes was performed by the average linkage hierarchical clustering method. According to criteria of the hybrid dynamicTreecut software package, each lncRNA network module had a minimum of 30 genes. After confirming the gene modules, we calculated the eigengenes of each module in turn, then clustered the modules, and merged the closer modules into new ones by setting height=0.25, deepSplit=2, and minModuleSize=30. We further analysed the functions of genes that were most correlated with the immune score in the modules. KEGG and GO enrichment analysis of the genes were performed with FDR <0.05 using R package clusterProfiler.

### Constructing the PPI network

We analysed PPI network of these 36 immune microenvironment-related genes using R package. We mapped the 36 genes from STRING database, selected interaction scores greater than 0, and then obtained the network relationship among these genes.

### Prognostic value of 12 immune microenvironment-related genes

To analyse the prognostic value of 12 immune microenvironment-related genes, all samples were divided into high and low expression groups according to their expression profiles. Then, prognostic differences between the two groups were analysed by the K-M method.

### External validation in GEO datasets and Human Protein Profiles

To ensure the accuracy of results from TCGA cohort, Gene Expression Omnibus (GEO, https://www.ncbi.nlm.nih.gov/geo/) datasets were analysed to verify the relationship between the 12 genes and immune scores. GSE62232, containing 81 liver cancer samples and 10 normal samples, was selected. The standardized expression matrix and sample information files were downloaded, and immune scores for each sample were calculated using R ESTIMATE package. We also extracted the gene expression profiles of the 12 immune-related genes, and then calculated Pearson’s correlation coefficient between the genes and the immune scores.

To further verify the accuracy of the results, we analysed the expression of the proteins encoded by the 12 immune-related genes using clinical specimens from The Human Protein Atlas (https://www.proteinatlas.org) [28].

The data from TCGA, GEO and The Human Protein Atlas were all publicly available and open access, so no approval was needed from the ethics committees.

### Quantitative real-time polymerase chain reaction (qRT-PCR) evaluation

From January 2017 to December 2018, 10 HCC patients who underwent curative resection and didn’t receive neoadjuvant therapy before surgery at the First Affiliated Hospital of Zhengzhou University (Zhengzhou, China) participated in this study in accordance with the provisions of Helsinki Declaration.

To evaluate the gene expression of *TMC8* and *BIN2*, 10 pairs of fresh HCC and adjacent normal samples were collected and tested using qRT-PCR method. Total RNA was extracted with Trizol reagent (Invitrogen, USA) and reverse-transcribed into cDNA using RevertAid First Strand cDNA Synthesis Kit (Thermo, USA) according to the manufacturer’s instruction. qRT-PCR was performed with the FastStart Universal SYBR Green Master (Rox) (Servicebio, China). The quantitative levels of gene expressions in HCC and adjacent paracancerous tissue were evaluated using the 2-ΔΔCt relative quantification method. The primers were as follows: TMC8: forward primer, 5′-GACTCTGCTGGGTCAGGGCTAT-3′, reverse primer, 5′-TCCACCTTGAACTCGTTGCTG-3′; BIN2: forward primer, 5′-ACGAGGAGAAACTGGCTGACC-3′, reverse primer, 5′-CACTGTCATAGTCCACGAGTTTCC-3′; β-actin (used as control): forward primer, 5′-GGAAGCTTGTCATCAATGGAAATC-3′, reverse primer, 5′-TGATGACCCTTTTGGCTCCC-3′.

### Western blotting analysis

Proteins were isolated from these 10 pairs of fresh-frozen HCC and adjacent normal tissues as previously described [29]. Protein concentrations of *TMC8*, *BIN2* and *SPOCK2* were measured using bicinchoninic acid assay. Equal amounts of proteins were separated by sodium dodecyl sulfate polyacrylamide gel electrophoresis (SDS-PAGE) and transferred to a polyvinylidene difluoride (PVDF) membrane (Millipore, USA). After blocking with 5% skimmed milk, the membrane was incubated with primary rabbit polyclonal antibodies (TMC8, BS-13116R, 1:500, Bioss, China; BIN2, BS-9727R, 1:500, Bioss, China; SPOCK2, AB217044, 1:1000, Abcam, UK) and ACTIN (GB11001, 1:3000, Servicebio, China) overnight at 4 °C, respectively. After washing the membrane, it was then incubated with a horseradish peroxidase-conjugated goat anti-rabbit secondary antibody (GB23303, 1:3000, Servicebio, China). Finally, the bound immunocomplexes were detected using ECL methods.

### Immunohistochemical (IHC) validation of the immune-related genes

All haematoxylin and eosin stained slides of tumor and adjacent non-tumour samples from another 35 HCC patients, confirmed by two experienced pathologists, were collected. Three immune-related genes, *TMC8*, *BIN2* and *SPOCK2*, were selected to be further validated with immunohistochemical method. All formalin-fixed paraffin-embedded HCC samples were collected to examine the protein levels of the three immune-related genes.

Slides with 4um sections from the paraffin-embedded specimens were deparaffinized and rehydrated. After conducting heat-mediated antigen retrieval, endogenous peroxidase activity was blocked by incubating the sections with 3% hydrogen peroxide at room temperature for 25 min. Then the slides were washed with phosphate buffered saline (PBS) three times and incubated with bovine serum. They were further incubated with primary and secondary antibodies. After washing with PBS and incubating with 3.3′-diaminobenzidine solution for 5 min, each section was counterstained with hematoxylin. All IHC slides were analyzed using Image-pro plus 6.0 software (Media Cybernetics, USA). The results were tested by two pathologists. For *TMC8*, *BIN2* and *SPOCK2* expression analysis, the relevant primary antibodies (BS-13116R, 1:400, Bioss, China; BS-9727R, 1:200, Bioss, China and AB217044, 1:200, Abcam, UK) were used. The results were all expressed with mean areal density (MAD).

### Statistical methods

All analyses were conducted using R software (version 3.5.2). ImmuneScore, StromalScore and ESTIMATEScore of each sample were calculated with R package ESTIMATE. The abundance of eight immune cell types, endothelial cells, and fibroblasts in each sample were estimated with MCPcounter software. Distances between each transcript were calculated with Pearson’s correlation coefficient. WGCNA was constructed using the R package WGCNA. The relationships between immune scores and HBV/HCV/molecular subtypes were tested by Analysis of Variance test. Survival R package was used for Kaplan-Meier curve analysis. Wilcox test was performed to analyze relationships between ESTIMATE immune scores and TP53, CTNNB1, AXIN1 mutations. Correlations between immune-related genes and immune checkpoint gene, ImmuneScores were studied by corrgram R package. The two-tailed paired t-test was used for data of *TMC8*, *BIN2* and *SPOCK2* expression at mRNA and protein level. All statistical tests were two sided and p values <0.05 were considered as statistically significant.

## Supplementary Material

Supplementary Figure 1

Supplementary File 1

Supplementary File 2

Supplementary File 3

Supplementary File 4

Supplementary File 5

Supplementary File 6
