# Supplementary material for: Prognostic values of immune scores and immune microenvironment-related genes for hepatocellular carcinoma
Source: Aging (Albany NY). 2020 Mar 25;12(6):5479–99. doi: 10.18632/aging.102971 (PMC7138561; doi:10.18632/aging.102971)
Supplement: Supplementary Figure 1 [file aging-12-102971-s007..pdf]

SUPPLEMENTARY FIGURE

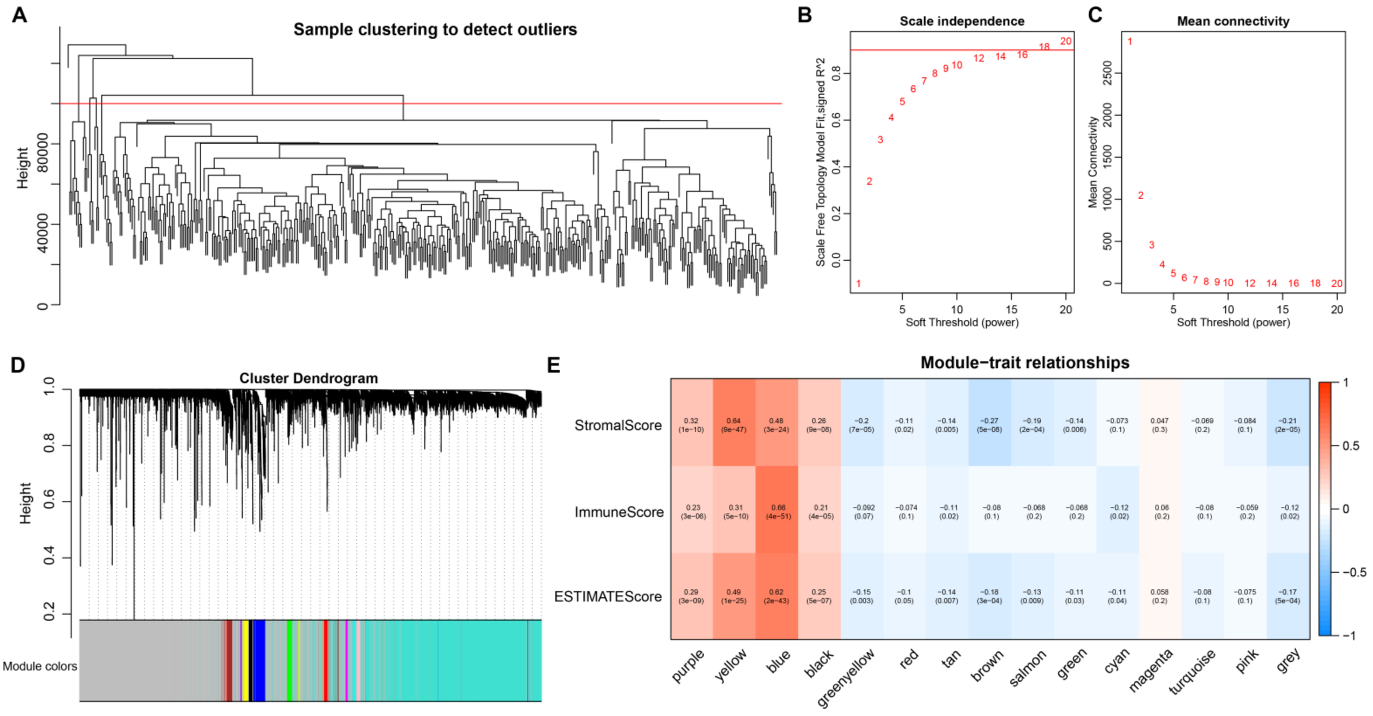

Supplementary Figure. (**A**) Clustering analysis of all samples. 396 samples finally obtained after deleting outliers with height larger than 100000. (**B**, **C**) Analysis of network topology for various soft-thresholding powers to ensure it being scale-free network. (**D**) Results of gene dendrogram and module colors. 15 modules were obtained. (**E**) Module-trait relationship between the 15 modules eigenvectors and the three immune scores.
